# Perspectives on Games, Computers, and Mental Health: Questions about Paradoxes, Evidences, and Challenges

**DOI:** 10.3389/fpsyt.2016.00122

**Published:** 2016-07-04

**Authors:** Martin Desseilles

**Affiliations:** ^1^Department of Psychology, University of Namur Medical School, Namur, Belgium; ^2^Clinique psychiatrique des frères Alexiens, Henri-Chapelle, Belgium

**Keywords:** games, computers, mental health, playing, learning, serious game, emotional regulation, critical period

## Abstract

In the field of mental health, games and computerized games present questions about paradoxes, evidences, and challenges. This perspective article offers perspectives and personal opinion about these questions, evidences, and challenges with an objective of presenting several ideas and issues in this rapidly developing field. First, games raise some questions in the sense of the paradox between a game and an issue, as well as the paradox of using an amusing game to treat a serious pathology. Second, games also present evidence in the sense that they involve relationships with others, as well as learning, communication, language, emotional regulation, and hedonism. Third, games present challenges, such as the risk of abuse, the critical temporal period that may be limited to childhood, their important influence on sociocognitive learning and the establishment of social norms, and the risk of misuse of games.

## Introduction

In the field of mental health, the electronic revolution, through the use of computers and the Internet, questions the concept of games. While the game is part of the psychological development of every individual, the electronic context leads to a redefinition of its use in mental health. This redefinition involves the identification of paradoxes regarding the use of games to deal with complex and serious problems. The identification of potential mechanisms of action underlying the use of games in mental health is also important to better identify targets for therapeutic intervention. Finally, the challenges, including variable concept of games, depending on gamers (kid games or adult games), and the possible misuse of information that can be made on the grounds that it is a game, also seem important to question. We will develop several ideas, including place of games in mental health and the idea of games both in the development of the child’s psyche and in the evaluation of adult relationships.

## Paradoxes

Questions arise from the paradox that playing a game should mean that there are no issues. Amusingly, in French, the word for “I” (*je*) is pronounced the same as the word for “game” (*jeu*), and the word for “issue” (*enjeu*) sounds very similar. When *issues* become too prominent, *I* am no longer playing a *game*, because there are risks, and risks come with consequences.

In addition, can we play with something as serious as mental health? A depression is no fun, and schizophrenia is even less fun. In this case, illness is certainly not a game, but games can be used as tools for communicating and regulating emotions in mental illness. However, although humor is a useful approach in mental health (1), it should not be confused with irony or cynicism. Cyberpsychology research is sometimes too “detached” from the concrete reality of mental illnesses and should not discredit the concrete stigma that follows mental illnesses when proposing computer games in the treatment of such serious illnesses.

If our main focus in this perspective is computer games, our reflections encompass certain aspects of games involving people. As such, it may appear an absence of a delimitation of “game” concepts. A taxonomy and description of games and their use in mental health has been proposed by recent researchers such as Miller (2) and would be far beyond the scope of our perspectives. Far from wanting to present the specific needs and applications of games in mental health, we wish to question some issues that this area can raise.

## Evidence

First, it is widely held that games are central to relationships between individuals and the “other,” to communication, and to the notion of ex-istence itself (to *ex-sist*: from Latin *ex-sisterer*, to “stand apart”). From Latin, *Ludus* (game in action) replaced *jocus* (game in speech) and absorbed its value (3). By doing so, we tend to forget that play and games are made of language and communication. Through games (for example, “peek-a-boo,” where the other is first hidden and then revealed, or the “bobbin” toy, which is set loose and then wound back in, or the game of “*fort/da*,” described by Freud as he watched his grandson make objects “disappear”), children experience disorientating surprises when something or someone becomes absent, followed by marvelous delight when the once absent other suddenly returns. Thus, games allow children to discover the permanence of connections with the other, or the continuity of the other as an object. The prominent psychotherapist Mélanie Klein and others later developed theories of the application of games and play to child psychanalysis (4). Importantly, in his book *Playing and Reality*, Winnicott states that “Playing is an experience, always a creative experience, and it is an experience in the space-time continuum, a basic form of living” [Ref. (5), p. 54]. Winnicott beautifully expresses the importance of play as an area that allows the interweaving movement between inner reality and the outside. Freud also stated that “The opposite of play is not seriousness but rather reality,” recognizing the seriousness of children’s play, creating an alternative to reality.

Second, games and play facilitate learning [e.g., Ref. (6), and also https://news.stanford.edu/news/2013/march/games-education-tool-030113.html] that is progressive, through trial and error, and without excessive guilt, because “it’s only a game.” When one plays a game, there is a desire to improve and make progress, there is no judgment, and the rules allow a group of players to assume delineated roles. The abovementioned homophony of the French words for “game” (*jeu*) and “I” (*je*) along with the closely resembling verb “play” (*joue*) reminds us that the “I” (or self) is at the center of the game: “*I play*” (*je joue*), therefore I learn.

Third, computers enable people to play games, among other things, but more to the point, they allow the environment to be redesigned and transformed. For example, the tempo of the presented stimuli can be changed to positively bias certain behaviors or images in order to correct biases of thought and memory [e.g., Ref. (7)]. If games are a primary mode of communication that existed before computers, the computer enables the addition of a network dimension (so that groups that are dispersed around the globe can play together), continuity (the game can be paused and picked up again later), singularity (individuals can play alone on a computer), diversity (a vast range of games are possible), precision (a variety of parameters can be measured, ranging from behaviors to players’ personalities), and so forth. The computer is therefore a tool that allows people to communicate with others (in real time or not) or with sets of users in a network (in real time or not). An interesting development of network gaming is crowdsourcing games that bridge the limits between games and issues, since they use the playing motivation and intelligence of people to solve real important scientific problems (8).

Fourth, the game is a *primary and primordial* form of communication among individuals, because even the youngest humans, babies, can interact with adults through play. They can not only play games like “catch me” and “hide and seek” but also engage in forms of informal play like tickling, attempting to do something again and again, trying out new words, and trying to achieve something. By learning how to verbalize, by using language and playing word games, babies become children (9). Games facilitate learning because they contain the critical elements of *repetition* as well as *surprise*. Thus, even when the players know a game very well, they can still be surprised. This feeling of surprise occurs during moments of full awareness and cognitive reevaluation, so that things can be seen in a new light, with or without interpretation. This helps children establish the bases for *emotional regulation* and fosters the acquisition of *emotional competencies* (10). Hence, the game is above all a means, or mode, of communication, which in metonymic terms means the object that enables communication. Adapting the classic model of Gross (11), we may propose that games involve (1) a choice or an intention to place oneself in a certain situation; (2) a direction of one’s attention or attentional deployment (what I am doing, what the other is doing); (3) cognitive reevaluation when questions arise or when the game changes as other players make progress; and (4) modulation of emotional responses, along with learning about the reactions of others when they are sad or happy for you or for themselves. As the game develops, these various factors are deployed in turn, and all of them contribute to mental health and well-being. Specifically, an European study has elegantly built a platform offering three mini-games that impact different components of emotion regulation as a complementary therapy tool in people with an impulsive disorder (12).

Fifth, further to the idea of emotional regulation, a hypothesis on the function of emotional regulation in dreams was proposed by Revonsuo, who suggests that dreams are a kind of *safe laboratory* where we can learn how to react appropriately to frightening stimulations (Threat Simulation Theory) (13). In games, as in dreams, even though appearances may closely resemble reality (and the computer enables pushing that resemblance much farther than ever before), the fact remains that there are no consequences. At least, this should be the case, or else it’s no longer a “game.” The game is therefore a safe laboratory for guessing what the other is thinking, to interact with the other, and so on. In this sense, the game is symbolic, and it fosters both *representation and mentalization*. Referring back to Freud, it facilitates a thicker preconscious. Beyond the metacognitive impact of game, concrete behavioral adaptation might occur since it is observed in animal games, which are without negative consequences and moreover deal with important aspect of their social and survival behaviors [e.g., Ref. (14)].

Sixth, a hedonistic approach [e.g., Ref. (15)] is beneficial for mental health insofar as it supports several temporally defined dimensions (past, present, and future) of memory retrieval, pleasure, and anticipation of shared times, without involving risk. Therefore, when applied to games, a hedonistic time perspective would also be beneficial for mental health. We stress this approach that as to be distinguished from pure rewarding approach, in which television serial or games with multiple level are especially experts, and that could induce more addictive stress than euphoric, serene and peaceful pleasure.

## Challenges

First, although video games can contribute to the well-being (16), spending long hours playing has been associated with a number of *harmful consequences* [e.g., Ref. (17)]: social retreat and withdrawal (“The only thing I do is play”; “I don’t care about anything else”), dependence (“I can’t stop playing”), depression (“I play all the time”; “That’s all I do, because nothing else interests me”), nervousness or anxiety (“I want to be able to do it”; “Can I do it?”; “I don’t know how to do anything else”), disrupted sleep patterns (“The computer screen keeps me awake”; “I’m chronically sleep-deprived”), and so on. But this is not new. Long before the computer, there were card-playing fanatics. However, the permanent availability (24/7) and the option of playing alone on a machine are certainly factors that distinguish the games of today from those of yesteryear, and that have turned gaming into a formidable challenge.

Second, for children, almost anything can be a “game,” because unlike adults, children are largely unaware of the issues associated with objects and events. Children can choose and ask for the toys that they want, and they willingly follow the rules that are imposed. And even if they ask “why” from time to time, the fact remains that games hardly ever inhibit their learning, in the non-pathological sense. At this developmental stage, disinhibition is not even a relevant term, because inhibition is only in the process of being established. However, compared with children, it is usually more difficult to persuade adults to play together. The inhibition that adults have established makes it harder for them to learn in this manner. Repeating a new word over and over is a game for a child, and possibly, a game for the adult who teaches it, but for most adults, word repetitions would be regarded more as performance than play. There appears to be a *critical temporal window* (i.e., childhood) during which play enables learning, whereas adults play less often, in the sense of child’s play. When adults reappropriate play in the form of serious gaming or educational games (gamification), they tend to be ambivalent about using a childish means for adult learning. In the expression, “Let’s stop playing and get serious,” the two terms “playing” and “serious” are opposed. Nevertheless, these terms are not necessarily antithetical, because games can be powerful learning tools. They use a multimodal approach to knowledge that incorporates combined sensoralities, emotions, and rational thinking. And, there is practically no inhibition involved, because the risks of the game are not the same as the risks that are incurred in serious work. Therefore, the conceptualization of certain developmental stages as critical stages that can be reactivated later on has implications for the learning process and for the conceptualization of mental health [e.g., Ref. (18)]. In addition, we have seen previously that “the opposite of play is not seriousness but rather reality” as Freud stated and further theorized by Klein and Winnicott. Thus, the oxymoron-like semantic construction “serious gaming” is questionable in more than one aspect. In such manner, the distinction drawn between games as tool for mental health and games as a potential risk for mental health might be considered cautiously since the concept of serious games is often misused or even abused. Nevertheless, the potential of serious games as mental health treatment has been very elegantly reviewed by Miller (2). Miller proposes a taxonomy that allows considering the many ways to include games in mental health care (2). We encourage the reader to refer to this review, since the aim of our perspective is more to question the notion of seriousness and reality, beyond the usefulness of recent and promising approaches using games for mental health.

Third, sociocognitive theories of learning by *imitation and identification*, first proposed by Bandura (19), contend that we identify with games, images, behaviors, and words. It is not that we see a model and then become that model. Instead, we learn about norms, standards, and status, which we then internalize, and which become important guidelines for distinguishing between the normal and the abnormal. Playing also involves thinking about “identities” and what we consider “normal” or “abnormal.” In this line of concern, there is a very important educational and social challenge to have games that reflect social and democratic values as well as pacific and respectful interactions, to cite only a few important components.

Fourth, at times, games involving people can be misused, in that they become overly *professionalized*. This has happened in music, tennis, football, and many other fields. Adults strive to coach their children, to push the game into the realm of professionalism. This can create discrepancies between the language of the child (play with no issues) and the language of the adult (issues with no play), if we may borrow the words of Ferenczi (20) and his “confusion of language.” When and how this transition occurs are some of the questions that we might raise here.

Fifth, in a further misuse of the playful spirit, games have also been repurposed as team-building exercises, whereby adults are brought together to create and nurture bonds. However, we know very well that these “games” are designed for observing the interactions that occur among the group, with the aim of improving these interactions and ultimately improving productivity. These games involve calculated monitoring by adult observers of other adults, who are aware that they are being observed, and who know that these observations may subsequently be used in their favor or disfavor. These are games “with issues,” largely used to investigate or generate group dynamics and their effects. This raises questions about the uses of imposed, monitored, and calculated games. Even if these uses of games involving people are accepted ways to work on professional bonds and even if people are aware of the consequences, it seems to us that spontaneity and unintentional curiosity seem to be far behind the scene of children’s gaming and constitute some specific characteristics of adult’s games.

## Conclusion

In conclusion, we wish to draw readers’ attention to the paradoxes, the issues, and challenges of the use of games in mental health, among others, in the context of the development of computers and the Internet. At the same time, important for the mental development of everyone, the games are a wonderful communication and emotion regulation tool, but must be framed in a critical way in order not to misuse it.

## Author Contribution

The author confirms being the sole contributor of this work and approved it for publication.

## Conflict of Interest Statement

The author declares that the research was conducted in the absence of any commercial or financial relationships that could be construed as a potential conflict of interest.

## References

[1] Galloway G Cropley A Humour and mental health: implications for psychotherapy. J Balt Psychol (2001) 2:5–14.

[2] Miller SM The Potential of Serious Games as Mental Health Treatment. University Honors thesis, Portland State University, Portland (2015).

[3] Rey A Dictionnaire historique de la langue française. Paris: Le Robert (2010).

[4] Klein M La psychanalyse des enfants. Paris: PUF, Quadrige Grands textes (2009).

[5] Winnicott DW Playing and Reality. London: Tavistock Publications (1971).

[6] Hedges JH Adolph KE Amso D Bavelier D Fiez JA Krubitzer L Play, attention, and learning: how do play and timing shape the development of attention and influence classroom learning? Ann NY Acad Sci (2013) 1292:1–20. 10.1111/nyas.12154 23763338 PMC3842829

[7] Desseilles M Pichon S Vuilleumier P Régulation des émotions et processus attentionnels. In: Mikolajczak M Desseilles M, editors. Traité de regulation émotionnelle Bruxelles. Bruxelles: De Boeck (2012). p. 103–116.

[8] Good BM Loguercio S Griffith OL Nanis M Wu C Su AI. The cure: design and evaluation of a crowdsourcing game for gene selection for breast cancer survival prediction. JMIR Serious Games (2014) 2(2):e7. 10.2196/games.3350 25654473 PMC4307816

[9] Berger FF De l’infans à l’enfant: les enjeux de la structuration subjective. Bull Psychol (2005) 58:505–12. 10.3917/bupsy.479.0505

[10] Mikolajczak M Desseilles M La régulation émotionnelle: historique, conceptualization et enjeux. In: Mikolajczak M Desseilles M, editors. Traité de régulation émotionnelle Bruxelles. Bruxelles: De Boeck (2012). p. 19–32.

[11] Gross JJ Emotion regulation: current status and future prospects. Psychol Inq (2015) 26:1–26. 10.1080/1047840X.2014.940781

[12] Fernández-Aranda F Jiménez-Murcia S Santamaría JJ Gunnard K Soto A Kalapanidas E Video games as a complementary therapy tool in mental disorders: PlayMancer, a European multicentre study. J Ment Health (2012) 21:364–74. 10.3109/09638237.2012.664302 22548300 PMC3433177

[13] Revonsuo A The reinterpretation of dreams: an evolutionary hypothesis of the function of dreaming. Behav Brain Sci (2000) 23:877–901. 10.1017/S0140525X00004015 11515147

[14] Smith JM The theory of games and the evolution of animal conflicts. J Theor Biol (1974) 47:209–21. 10.1016/0022-5193(74)90110-6 4459582

[15] Sailer U Rosenberg P Nima AA Gamble A Gärling T Archer T A happier and less sinister past, a more hedonistic and less fatalistic present and a more structured future: time perspective and well-being. PeerJ (2014) 11:e303. 10.7717/peerj.303 24688878 PMC3961480

[16] Jones CM Scholes L Johnson D Katsikitis M Carras MC. Gaming well: links between videogames and flourishing mental health. Front Psychol (2014) 5:260. 10.3389/fpsyg.2014.00260 24744743 PMC3978245

[17] Zamani E Chashmi M Hedayati N. Effect of addiction to computer games on physical and mental health of female and male students of guidance school in city of Isfahan. Addict Health (2009) 1:98–104. 24494091 PMC3905489

[18] Hensch TK Bilimoria PM. Re-opening windows: manipulating critical periods for brain development. Cerebrum (2012) 2012:11. 23447797 PMC3574806

[19] Bandura A Psychological Modelling. New York: Lieber-Antherton (1971).

[20] Ferenczi S Sprachverwirrung zwischen den Erwachsenen und dem Kind. Die Sprache der Zärtlichkeit und der Leidenscha#. Exposé fait au XIIème Congrès International de Psychanalyse à Wiesbaden. Internationale Zeitschrift für Psychoanalyse, Herausgegeben von Sigm. Freud. XIX. Band, 1933, Heft 1/2 (1932).

